# Placenta Accreta: A Case Report on the Role of Interventional Radiology

**DOI:** 10.7759/cureus.47680

**Published:** 2023-10-25

**Authors:** Mafalda Machado, Teresa Dionísio, Diogo Rocha, Marta Campos, Pedro Sousa

**Affiliations:** 1 Radiology, Centro Hospitalar Universitário do Algarve, Faro, PRT; 2 Radiology, Centro Hospitalar Vila Nova de Gaia-Espinho, Vila Nova de Gaia, PRT; 3 Obstetrics and Gynaecology, Centro Hospitalar Vila Nova de Gaia-Espinho, Vila Nova de Gaia, PRT

**Keywords:** pregnancy, interventional, radiology, balloon occlusion, placenta accreta

## Abstract

Placenta accreta spectrum disorder is a pregnancy-related disorder responsible for important post-partum morbimortality, associated with intractable or massive hemorrhage, leading to uterine loss in up to 64% of women. Despite international recommendations advocating planned preterm cesarean hysterectomy for the management of these patients, uterus preservation management is being continuously reported with the implementation of minimally invasive bleeding reduction strategies, such as prophylactic balloon-assisted occlusion. We present the case of a 40-year-old pregnant woman with a previous cesarean, diagnosed with placenta previa and suspected placenta accreta on magnetic resonance after having second-trimester vaginal bleeding. A peri-operative multidisciplinary panel was involved, in collaboration with the interventional radiologist, and the c-section was scheduled for 36 weeks of gestation. The prophylactic balloon-assisted occlusion was successfully performed, minimizing the blood loss and allowing a uterus-preserving approach.

## Introduction

The placenta accreta spectrum (PAS) is an abnormal placental invasion of the myometrium or extrauterine structures and comprises placenta accreta, placenta increta, and placenta percreta, depending on the depth of the myometrial invasion, from the least severe to the most severe types, respectively [1,2]. Its prenatal diagnosis is of the most importance due to the increased risk of life-threatening perinatal bleeding, which is the cause of maternal mortality in seven percent of cases [1-3].

The main risk factors are a previous cesarean and placenta previa, the latter corresponding to an abnormally low-lying placenta in proximity or covering the internal cervical os. Due to the increasing number of cesarean deliveries worldwide, which is expected to become more pronounced in the years to come, the incidence of placenta accreta has increased in the last decades [3,4].

A multidisciplinary team of professionals is crucial to the management of these patients, including obstetrics, urology, radiology, anesthesiology, and blood banking, in order to reduce maternal mortality [3]. The recommended approach is a preterm cesarean hysterectomy [1]. However, in recent years, some bleeding reduction strategies, such as prophylactic balloon-assisted occlusion (PBAO), have been implemented to obtain the best results and even perform uterus-sparing deliveries. PBAO consists of placing occlusion balloons (OBs) in the internal iliac arteries before the planned cesarean and inflating them immediately after the delivery to reduce post-partum bleeding. It has been proven to reduce the rate of blood loss in patients with placenta accreta spectrum (PAS) and decrease the rate of hysterectomy compared to those without it [5,6].

## Case presentation

**Figure 1:**
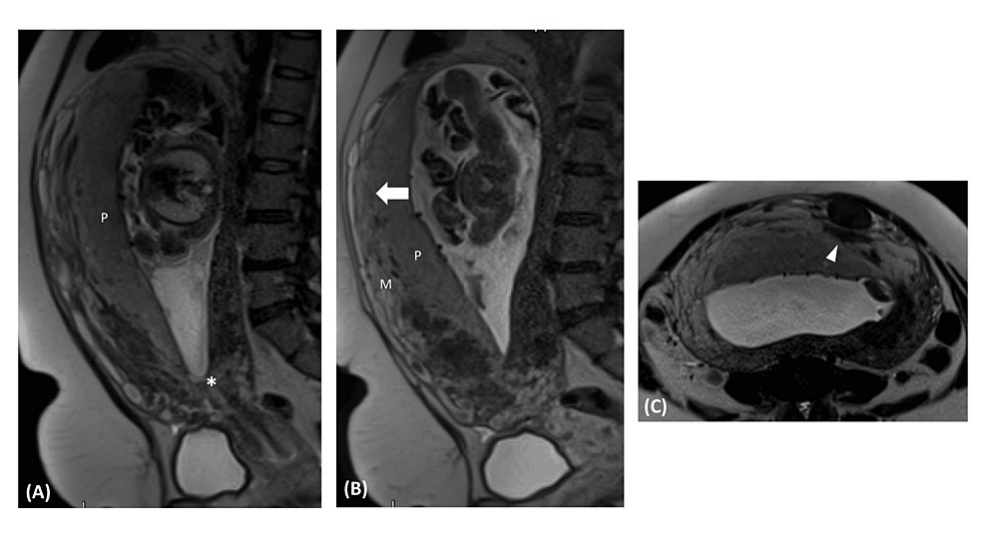
Fetal MRI. (A) Sagittal T2 single-shot fast spin echo (SSFSE) shows anterior placenta (P) in an abnormally low-lying position, distancing 1.4 cm from internal cervical os (*), diagnosing placenta previa. (B) and (C) Sagittal and axial T2 SSFSE reveal additional findings suggestive of placentary invasion of the myometrium (M), such as in definition or thinning of retroplacental T2 dark border (large arrow in B) and dark bands (arrowhead).

A 40-year-old woman presented to the emergency room with vaginal bleeding at 27 weeks of gestation. She had a previous cesarean delivery and obesity as a relevant personal history. She was admitted for vigilance and performed an ultrasound and MRI, which diagnosed the placenta previa and placenta accreta (Figure 1).

The preterm cesarean was planned for the 36th week of gestation, in collaboration between the obstetrician, interventional radiologist, anesthesiologist, and immunohemotherapist.

**Figure 2:**
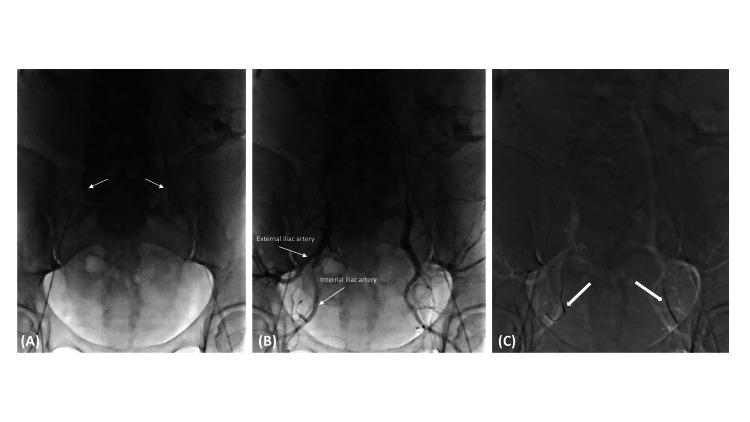
Prophylactic balloon-assisted occlusion. Fluoroscopy at the angiography room before the scheduled cesarean. (A) After bilateral femoral access, both common iliac arteries and internal iliac arteries were canulated using a 5F Cobra-1 catheter (arrows) and a 0.035'' hydrophilic angled-tip guidewire, by two interventional radiologists simultaneously. (B) Iodinated contrast injection was used to serve as a roadmap and confirm the correct position. (C) The occlusion balloons were positioned over-the-wire in the appropriate location (internal iliac arteries–bold arrows) and were tested by inflating until nominal pressure was achieved, one at a time.

On the due day, the patient went to the angiography room (AR) for the placement of OB in the internal iliac arteries. The anesthesiologist performed an epidural anesthesia. After local anesthesia with 2% lidocaine, bilateral femoral access was achieved using 7F sheaths. Both IIA were cannulated using a contralateral approach, by two interventional radiologists simultaneously to reduce radiation exposure, using a 5F Cobra-1 catheter (Glidecath®, 5F, 100cm, Terumo) and a 0.035'' hydrophilic angled-tip guidewire (Glidewire® Hydrophilic Coated Guidewire, 0.035'', 180 cm, Terumo). Contrast injection was used to serve as a roadmap (Figure 2). The OBs were placed over-the-wire in the internal iliac arteries, immediately after common iliac artery bifurcation. We used 6F-compliant occlusion balloon catheters (Berenstein Occlusion Balloon® 8.5 x 11.5 mm, 0.038'', Boston Scientific). Both balloons were tested by inflating until nominal pressure was achieved, one at a time, to avoid fetal hypoxia. One of the balloons was defective and had to be replaced.

During these steps, fluoroscopic time was kept to a minimum, and radiation dose reduction strategies were implemented, such as last-image hold instead of digital subtraction angiography. Additionally, the X-ray tube was kept as far as possible from the patient, and the detector was kept as close as possible. Total fluoroscopy time was 7.2 minutes, dose area product (DAP) was 5968 µGym^2^ and air kerma was 222 mGy.

After correct positioning of the OB, the sheaths were sutured to the skin, and both sheaths and OB were attached to the skin with adhesive, to prevent their displacement. The patient was moved to the operating room (OR), and the OB was attached to the inflation pumps and correct positioning was confirmed by fluoroscopy. The obstetricians then proceeded with the cesarean surgery.

After fetus extraction, both OBs were inflated to their nominal pressure. An attempt to remove the placenta was successful, and no active bleeding was depicted, allowing a uterine-preserving surgery. The patient was again transferred to the AR, and the balloons were deflated, with a total inflation time of two hours. Contrast media injection showed no signs of active bleeding, and the balloons and sheaths were removed. The puncture sites were closed with manual pressure.

During the procedure, one pack of red blood cells was transfused. The newborn was healthy, weighted 2455 g and had an Apgar Score (activity, pulse, grimace, appearance, and respiration) of four, eight, and nine at the first, fifth, and tenth minutes, respectively. The lowest hemoglobin value was 6.8 g/dL, and three packs of blood cells were needed during the hospitalization. A pseudoaneurysm of the left common femoral artery was detected in a computed tomography (CT) performed two days after the procedure, which was successfully treated with percutaneous thrombin injection. The patient was discharged on the seventh day post-partum. A two-year follow-up consultation revealed no complications or significant disability.

## Discussion

PBAO is a rising procedure proven to be an effective adjunctive technique in reducing post-partum bleeding and potentially preventing hysterectomy in women with placenta accreta spectrum [7]. Possible complications of the procedure are rare and involve arterial or venous thrombosis, complications associated with the arterial access, such as hematoma and pseudoaneurysm, as in the case presented, and complications associated with incorrect balloon inflation, such as artery rupture and dissection [1,7]. To minimize these risks, balloon dilatation should be as short as possible, with a maximum duration of intermittently inflated IIA balloons of about four hours. A low threshold for angiography or CT is important for the early detection and management of complications [1,8].

There is concern regarding the radiation exposure of the fetus during these procedures. However, it has been proven to be low when dose reduction techniques are implemented, resulting in low fetus-absorbed dose [9].

PBAO was proven to be a safe ally for the management of these women and interventional radiologists should be an integrating part of a multidisciplinary care team of experienced specialists [6,8].

## Conclusions

This case report demonstrates the potential role of PBAO as an adjunctive technique to programmed cesarean delivery, preventing post-partum hemorrhage and preserving the uterus in women with placenta accreta spectrum. It is a safe procedure for women and their fetuses when performed by experienced interventional radiologists. We hope this case report can provide technical information for those intending to implement this procedure.
